# Association of organizational and patient behaviors with physician well-being: A national survey in China

**DOI:** 10.1371/journal.pone.0268274

**Published:** 2022-05-31

**Authors:** Xiaoyu Wang, Yimei Zhu, Fang Wang, Yuan Liang

**Affiliations:** 1 Department of Social Medicine and Health Management, School of Public Health, Tongji Medical College, Huazhong University of Science and Technology, Wuhan, China; 2 School of Media, Communication and Sociology, University of Leicester, Leicester, United Kingdom; 3 Department of Epidemiology and Health Statistics, School of Health, Wuhan University, Wuhan, China; University of Nebraska Medical Center, UNITED STATES

## Abstract

This study aims to investigate the association of organizational and patient behaviors (reflecting the internal and external environment of hospital, respectively) with physician well-being. A national cross-sectional survey was conducted in 77 hospitals across seven provinces in China between July 2014 and April 2015. Physician well-being was assessed with job satisfaction, career regret and happiness. Organizational behaviors were assessed with organizational fairness, leadership attention and team interaction; patient behaviors were assessed with patient trust and unreasonable requests from patients. Of a study sample of 3,159 physicians, 1,788 were men (56.6%) and 1,371 were women (43.4%). Overall, positive organizational and patient behaviors reported by physicians were relatively low. Negative organizational behaviors and patient behaviors including lower organizational fairness, lower leadership attention, lower team interaction and lower patient trust were associated with lower job satisfaction and lower life satisfaction, and higher career regret. The association between organizational behaviors and physician well-being exhibited some gender differences, while no clear gender difference was found for the relationship between patient behaviors and physician well-being. Given the importance of physician well-being for the healthcare system, interventions for improving internal and external hospital environments (e.g., organizational fairness, leadership attention, team interaction and patient trust) may benefit physician well-being.

## Introduction

Physician well-being is particularly important in the healthcare system, including its impact on physicians’ own physical and mental health and career development [1–5], as well as the quality of medical services and patient satisfaction [6–9]. Studies on improving physician well-being revealed various underlying factors that may provide benefits. While some studies have emphasized the importance of individual intrinsic motivational factors [10–12], others have suggested that [6, 13], faced with widespread physicians’ distress, individual susceptibility (individual factors) may not be primary, and environmental factors (including the internal and external hospital environments) may be more important.

Most research on the internal hospital environments has focused on working environment factors [14–19], including workload, workflow, job autonomy, hospital teaching attributes, practice characteristics, scheduling issues, leadership behaviors, use of electronic health records, number of doctors (or nurses) per ward bed, average daily admissions, and average occupancy workload. In fact, factors such as job autonomy, hospital teaching attributes, practice characteristics, and scheduling issues are difficult for healthcare providers (including clinicians and hospitals/organizations) to modify [20, 21] because the internal hospital environments are broad in scope and relatively complex to examine. Compared to the structural factors of organization, organizational behaviors (e.g., organizational fairness, leadership attention, and team interaction) may be more feasible to modify. In terms of the external hospital environment including the social environment, previous research [22, 23] has focused on financial compensation, and external competition, and less on the attitudes and behaviors of patients and their families. Although previous studies have incorporated patient factors into working conditions for physicians and hospitals [6,10], patients are situated in a societal context and are affected by social environmental factors outside the hospital. Physicians have closer contact with patients in their everyday work environment than those who they receive incentives or penalties from such as medical insurance agencies; hence factors related to patients may be more relevant to improve physician well-being. In addition, some previous studies [24–27] have focused on the associations between leadership behaviors and physicians’ burnout and satisfaction, however, the types of organizational behaviors are relatively simple [24, 25, 27, 28], such as leadership and/or organization fairness, and few explanatory variables were accounted for, lacking some organizational characteristics (e.g., specialties, teaching nature, and the ratio of physicians to beds) and physicians’ family factors, etc.

The present study used data from a national survey of physicians in China to explore the association of organizational and patient behaviors (reflecting the internal and external environment of hospital, respectively) with physician well-being.

## Methods

### Study design and participants

We conducted a survey between July 2014 and April 2015 using stratified cluster sampling strategy. Briefly, seven provinces were selected from each geographical area, with two from each of East and West China (Shandong and Jiangsu, and Gansu and Yunnan), and one each from South, Central and North China (Guangdong, Hubei and Beijing metropolis). The details of this survey have been described in a previous report [29], and there was a total of 85 eligible hospitals in the selected regions with 77 hospitals (90.60%) agreeing to participate. In each hospital, convenience sampling was used to select four surgical departments and four internal medicine departments (excluding obstetrics and pediatrics). A total of 528 departments were selected and all of their full-time physicians (n = 5,754) were eligible and invited to participate in the survey. Trained survey interviewers sent copies of the questionnaire) to each department, along with an explanation of the survey purpose and method. After one or two days, the interviewers returned to collect completed questionnaires. Participation was voluntary, and all data was kept confidential. Finally, 4,281 (74.4% response rate) physicians responded to the survey and 634 (11.0%) invalid questionnaires were excluded. The final valid responses were 3,159 (54.9%) after excluding 488 (8.5%) responses with incomplete information (S1 Fig in S1 File).

All participants provided oral informed consent for interviews. We obtained ethical approval from the institutional review board at the Tongji Medical College, Huazhong University of Science and Technology (Wuhan, China) [No. IORG0003571].

### Data collection

Exposure factors include three specific aspects of organizational behaviors (organizational fairness, leadership attention, team interaction) and two aspects of patient behaviors (patient trust and unreasonable requests from patients). One of the commonly used measurement for organizational behaviors is Colquitt’s Organizational Justice Scale [30, 31], which contains 4 dimensions and 20 items, although the length of this questionnaire limits its feasibility in nationwide studies. Based on the literature and expert consultation [32–36], we adopted similar survey indicators and methods in the current study. Organizational fairness was assessed using two single-item measures adapted from the full Colquitt’s Organizational Fairness Scale: pay fairness (reflecting distributional fairness), and task fairness (reflecting procedural fairness). Similarly, leadership attention was assessed using two single-item measures: interests attention (reflecting attention to physicians’ material needs) and opinions attention (reflecting attention to physicians’ emotional needs). Team interaction [37] was assessed using two single-item measures: number of dinners with colleagues per month (reflecting social interaction) and number of clinical case discussions with colleagues per month (reflecting work interaction). Unlike some prior studies [38–40], patient behaviors in this study were measured from the physicians’ perspective as two items of patient trust (intrinsic behavior) and unreasonable requests from patients (explicit behavior) were used as measures for patient behaviors. Each question was answered on a 5-point Likert scale and we recoded each question into four categories (see Table 2).

There were three outcome variables: job satisfaction, career regret and happiness. In reference to prior studies [11], three outcome variables were assessed using three single-item measures respectively in the current study. Job satisfaction was examined using the question “Overall, how would you rate your satisfaction with your work?”. Career regret was examined with the question “If you had an opportunity to choose your profession, would you become a physician again?” and the response was reverse coded to measure the degree of “regret”. Happiness was assessed with the question “Overall, what do you think your happiness score is?” and the response for happiness was reverse-coded as categorical variables (80–100 = “very high”, 60–79 = “higher”, 40–59 = “average”, 20–39 = “lower” and 0–19 = “very low”). Each question was answered using a 5-point Likert scale with response options ranging from “very low” to “very high”. Finally, all responses for the three outcome variables were recoded as binary variables: low (“very low/lower/average”) versus high (“higher/very high”).

In addition, we measured a number of factors previously reported to be associated with well-being of physicians, including socio-demographics (age, gender, marital status, education level, economic status and professional ranking), hospitals and department characteristics (hospital level, hospital type, academic status, physician specialty and the ratio of physicians to beds) and family support. Data of hospitals and department characteristics was obtained from the unit heads.

### Statistical analysis

To adjust for nonresponses, the data was weighted by respondents’ age and gender, according to the hospitals’ demographic information issued by the National General Hospital in 2015.

For crude comparisons, we used chi-square tests or Kruskal-Wallis tests for categorical variables and used binary logistic regression models to examine the association of organizational and patient behaviors with physician well-being. Given that women are considered having more family care responsibility whilst work obligations negatively impact family care roles [41, 42], there may be a gender difference for the impact of organizational and patient behaviors on physician well-being; hence the analysis of this study did a gender stratification. Two-sided tests were used for all the analyses, and *P* values of 0.05 or less were considered statistically significant. All analyses were performed using SPSS, version 22.0 (SPSS Inc., Chicago, IL, USA).

## Results

The primary characteristics of participants, departments and hospitals were summarized in Table 1. Among 3,159 respondents, 1788 were men (56.6%) and 1,371 were women (43.4%). More than 80% of physicians were married and less than 10% reported good financial conditions. In terms of gender difference, females were less likely to be surgeons than males (vs 25.8% vs 63.3%, *P*<0.001).

**Table 1:** The primary characteristics of participants, hospitals and departments.

	Men(n = 1788)	Women(n = 1371)	*P* Value
**Socio-demographics**			
Age, y			
≤34	519(29.0)	398(29.0)	<0.001
35–44	620(34.7)	476(34.7)	
≥45	649(36.3)	498(36.3)	
Marital status			
Single/Other	252(14.7)	254(19.1)	0.001
Married	1466(85.3)	1076(80.9)	
Education level			
Undergraduate and below	893(50.7)	739(55.9)	0.010
Master	636(36.1)	464(35.1)	
Doctor	234(13.3)	120(9.1)	
Economic status			
Very poor	227(12.7)	183(13.4)	0.889
Poor	392(22.0)	251(18.4)	
Average	1011(56.6)	828(60.6)	
Good	155(8.7)	105(7.7)	
Professional ranking			
Primary/Other	374(23.0)	300(25.6)	<0.001
Intermediate	490(30.2)	397(33.8)	
Senior	761(46.8)	477(40.6)	
**Hospitals and departments characteristics**			
Hospital level			
Secondary Hospital	255(14.3)	207(15.1)	0.509
Tertiary Hospital	1533(85.7)	1164(84.9)	
Hospital type			
Traditional Chinese Medicine	470(26.3)	413(30.1)	0.017
Western Medicine	1318(73.7)	958(69.9)	
Academic status			
No	1402(78.4)	1134(82.7)	0.003
Yes	386(21.6)	237(17.3)	
Physician specialty			
Internal medicine	656(36.7)	1018(74.3)	<0.001
Surgery	1132(63.3)	353(25.8)	
The ratio of physicians to beds			
<0.20	473(26.8)	389(28.9)	0.020
0.20–0.30	777(44.0)	485(36.1)	
≥0.30	517(29.3)	471(35.0)	
**Family support**			
Very small/Small	71(4.0)	37(2.7)	0.023
Average	330(18.5)	215(15.7)	
Large/Very large	1384(77.5)	1118(81.6)	

Table 2 showed the characteristics of physicians’ self-perceived organizational and patient behaviors. Overall, the rates of positive responses regarding organizational and patient behaviors reported by physicians were relatively low. Less than 10% of physicians reported good pay fairness and large interests attention from leaders. More than 70% of physicians had only 0–1 dinner with colleagues per month and 30–40% of physicians had ≥4 clinical case discussions with colleagues per month. Less than 15% reported high patient trust. Concerning gender difference, females were more likely to report fewer dinner with colleagues per month than males (70.4% vs 79.7%, *P*<0.001) and clinical case discussions (19.7% vs 29.0%, *P*<0.001).

**Table 2:** The characteristics of physicians’ self-perceived organizational and patient behaviors.

	Men(n = 1788)	Women(n = 1371)	*P* Value
Organizational behaviors			
**Organizational fairness**			
Pay fairness			
Very poor	533(29.8)	359(26.2)	<0.001
Poor	504(28.2)	374(27.3)	
Average	619(34.6)	512(37.4)	
Good/Very good	131(7.3)	124(9.1)	
Task fairness			
Very poor	295(16.5)	188(13.7)	0.002
Poor	388(21.7)	295(21.5)	
Average	877(49.1)	696(50.8)	
Good/Very good	227(12.7)	191(13.9)	
**Leadership attention**			
Interests attention			
Very small	595(33.3)	409(29.8)	0.009
Smalle	413(23.1)	334(24.4)	
Average	606(33.9)	513(37.4)	
Large/Very large	172(9.6)	115(8.4)	
Opinions attention			
Very small	634(35.5)	439(32.0)	0.020
Smalle	419(23.5)	337(24.6)	
Average	542(30.3)	485(35.4)	
Large/Very large	192(10.7)	110(8.0)	
**Team interaction**			
The number of dinners with colleagues per month			
≥4times	147(8.3)	80(5.9)	<0.001
3times	101(5.7)	53(3.9)	
2times	279(15.76)	145(10.6)	
0-1time	1255(70.4)	1090(79.7)	
The number of clinical case discussions per month			
≥4times	831(46.7)	460(33.8)	<0.001
3times	273(15.3)	215(15.8)	
2times	327(18.4)	290(21.3)	
0-1time	350(19.7)	395(29.0)	
Patient behaviors			
**Patient trust**			
Very low	267(15.0)	174(12.8)	0.642
Lower	497(27.9)	409(30.1)	
Average	801(45.0)	645(47.4)	
Higher/Very high	214(12.0)	133(9.8)	
**Unreasonable requests from patients**			
A lot	112(6.3)	80(5.8)	0.031
More	477(26.7)	412(30.1)	
Average	601(33.7)	470(34.3)	
Less/Rarely	595(33.3)	408(29.8)	

Table 3 reported gender differences on job satisfaction, career regret and level of happiness. More than half of physicians felt high level of happiness, however, physicians were not optimistic about their work, with low job satisfaction (30%–40%) and high career regret (nearly 70%). Additionally, differences in level of happiness between men and women were observed. Females reported higher level of happiness than males(54.6% vs 51.9%, *P*<0.05).

**Table 3:** Job satisfaction, career regret and happiness of physicians by gender.

	Men(n = 1788)	Women(n = 1371)	*P* Value
**Job Satisfaction**			
Very low	157(8.8)	93(6.8)	0.314
Lower	176(9.8)	153(11.2)	
Average	810(45.3)	601(43.8)	
Higher	506(28.3)	372(27.1)	
Very High	139(7.8)	152(11.1)	
**Career Regret**			
Very low	61(3.4)	36(2.6)	
Lower	142(7.9)	134(9.8)	0.686
Average	394(22.0)	288(21.0)	
Higher	322(18.0)	254(18.5)	
Very High	869(48.6)	659(48.1)	
**Happiness**			
Very low	198(11.1)	130(9.5)	
Lower	230(12.9)	141(10.3)	0.002
Average	432(24.2)	352(25.7)	
Higher	684(38.3)	517(37.7)	
Very High	244(13.7)	231(16.9)	

Table 4 summarized the association of organizational and patient behaviors with physician well-being after adjusting for all other explanatory variables. In general, patient behaviors were more significantly associated with physician well-being than organizational behaviors. In terms of patient behaviors, lower patient trust was associated with lower job satisfaction (OR _total_ = 0.22, 95% CI: 0.15–0.33, OR _men_ = 0.21, 95% CI: 0.12–0.36, OR _women_ = 0.18, 95% CI: 0.09–0.37) and lower level of happiness (OR _total_ = 0.32, 95% CI: 0.21–0.49, OR _men_ = 0.34, 95% CI: 0.19–0.58, OR _women_ = 0.22, 95% CI: 0.10–0.48) and higher career regret (OR _total_ = 5.32, 95% CI: 3.34–8.46, OR _men_ = 4.99, 95% CI: 2.75–9.07, OR _women_ = 8.20, 95% CI: 3.68–18.24). With regards to organizational behaviors, lower pay fairness was positively associated with higher career regret (OR _total_ = 2.80, 95% CI: 1.74–4.51, OR _men_ = 2.85, 95% CI: 1.48–5.49, OR _women_ = 3.07, 95% CI: 1.44–6.54); lower task fairness was positively associated with lower level of happiness (OR _total_ = 0.43, 95% CI: 0.28–0.67, OR _men_ = 0.42, 95% CI: 0.23–0.75, OR _women_ = 0.41, 95% CI: 0.21–0.82) and more frequent clinical case discussions with colleagues per month was positively associated with higher job satisfaction (OR _total_ = 1.46, 95% CI: 1.15–1.86, OR _men_ = 1.43, 95% CI: 1.02–1.99, OR _women_ = 1.68, 95% CI: 1.15–2.46).

**Table 4:** Multivariable logistic regression results for association of organizational and patient behaviors-related effects for physicians well-being.

	Job Satisfaction			Career Regret			Happiness		
	Total OR(95%CI)	Men OR (95%CI)	Women OR 95%CI)	Total OR(95%CI)	Men OR(95%CI)	Women OR(95%CI)	Total OR(95%CI)	Men OR(95%CI)	WomenOR(95%CI)
Organizational behaviors									
**Organizational fairness**									
Pay fairness									
Very poor	1.44(0.91,2.28)	1.25(0.66,2.37)	1.53(0.75,3.13)	2.80(1.74,4.51)***	2.85(1.48,5.49)**	3.07(1.44,6.54)**	0.88(0.54,1.44)	0.64(0.32,1.26)	1.41(0.67,2.96)
Poor	0.91(0.59,1.40)	0.86(0.47,1.57)	0.92(0.47,1.83)	2.85(1.84,4.39)***	2.13(1.19,3.83)*	4.70(2.30,9.58)***	0.88(0.55,1.40)	0.61(0.32,1.15)	1.35(0.67,2.75)
Average	1.13(0.75,1.69)	1.49(0.85,2.60)	0.76(0.40,1.44)	1.99(1.34,2.98)***	1.46(0.85,2.50)	3.16(1.62,6.15)***	0.88(0.57,1.36)	0.74(0.40,1.35)	1.00(0.51,1.98)
Good/Very good	1.00	1.00	1.00	1.00	1.00	1.00	1.00	1.00	1.00
Task fairness									
Very poor	0.50(0.33,0.75)***	0.36(0.21,0.63)***	0.71(0.36,1.41)	1.24(0.77,2.00)	1.24(0.65,2.34)	1.11(0.50,2.46)	0.43(0.28,0.67)***	0.42(0.23,0.75)**	0.41(0.21,0.82)*
Poor	0.68(0.47,0.91)***	0.65(0.40,1.05)	0.66(0.36,1.20)	1.09(0.75,1.59)	1.33(0.80,2.19)	0.79(0.41,1.51)	0.52(0.36,0.76)***	0.53(0.33,0.88)*	0.49(0.27,0.87)*
Average	0.60(0.44,0.83)***	0.47(0.31,0.71)***	0.86(0.51,1.44)	0.98(0.71,1.35)	1.21(0.79,1.84)	0.65(0.37,1.13)	0.73(0.52,1.02)	0.64(0.41,1.00)*	0.89(0.53,1.52)
Good/Very good	1.00	1.00	1.00	1.00	1.00	1.00	1.00	1.00	1.00
**Leadership attention**									
Interests attention									
Very small	0.43(0.27,0.67)***	0.66(0.37,1.18)	0.20(0.09,0.42)***	1.54(0.95,2.49)	0.73(0.38,1.38)	4.73(2.03,11.04)***	0.74(0.46,1.18)	0.80(0.43,1.48)	0.67(0.31,1.47)
Smalle	0.46(0.31,0.70)***	0.57(0.33,0.98)*	0.29(0.14,0.60)***	1.76(1.16,2.69)	0.89(0.51,1.56)	5.06(2.37,10.77)***	0.83(0.54,1.28)	1.00(0.57,1.76)	0.62(0.30,1.30)
Average	0.55(0.38,0.79)***	0.71(0.44,1.14)	0.34(0.18,0.65)***	1.20(0.83,1.74)	0.66(0.41,1.08)	3.41(1.74,6.67)***	0.87(0.59,1.29)	0.94(0.57,1.56)	0.76(0.39,1.48)
Large/Very large	1.00	1.00	1.00	1.00	1.00	1.00	1.00	1.00	1.00
Opinions attention									
Very small	1.19(0.75,1.87)	1.05(0.58,1.87)	1.29(0.59,2.84)	1.35(0.84,2.18)	2.29(1.24,4.24)**	0.73(0.30,1.74)	0.86(0.53,1.37)	0.95(0.52,1.75)	0.62(0.27,1.40)
Smalle	1.09(0.71,1.66)	1.18(0.69,2.03)	0.83(0.39,1.74)	0.92(0.60,1.40)	1.35(0.78,2.31)	0.52(0.24,1.14)	1.09(0.70,1.70)	1.16(0.66,2.03)	0.87(0.40,1.89)
Average	1.35(0.92,1.97)	1.30(0.81,2.10)	1.22(0.62,2.39)	0.80(0.65,1.17)	1.04(0.65,1.67)	0.51(0.25,1.04)	1.03(0.69,1.55)	1.21(0.73,2.00)	0.74(0.36,1.52)
Large/Very large	1.00	1.00	1.00	1.00	1.00	1.00	1.00	1.00	1.00
**Team interaction**									
The number of dinners with colleagues per month									
≥4times	0.69(0.48,1.00)**	0.95(0.61,1.48)	0.37(0.18,0.77)**	0.58(0.40,0.83)***	0.49(0.32,0.77)**	0.78(0.39,1.56)	1.48(1.03,2.14)***	2.07(1.32,3.25)**	0.67(0.35,1.30)
3times	1.30(0.86,1.97)	1.05(0.62,1.76)	2.28(1.07,4.85)	0.89(0.58,1.35)	1.01(0.60,1.69)	0.65(0.31,1.38)	1.89(1.21,2.95)***	1.86(1.08,3.21)*	2.57(1.10,6.02)*
2times	0.80(0.61,1.05)	0.95(0.68,1.32)	0.61(0.36,1.02)	0.96(0.73,1.26)	0.99(0.70,1.40)	0.89(0.54,1.47)	1.35(1.03,1.76)***	1.26(0.90,1.75)	1.57(0.96,2.57)
0-1time	1.00	1.00	1.00	1.00	1.00	1.00	1.00	1.00	1.00
The number of clinical case discussions per month									
≥4times	1.46(1.15,1.86)***	1.43(1.02,1.99)*	1.68(1.15,2.46)**	0.98(0.76,1.27)	1.13(0.80,1.61)	0.85(0.57,1.27)	0.93(0.73,1.18)	0.85(0.61,1.19)	1.08(0.74,1.56)
3times	1.10(0.82,1.47)	1.42(0.94,2.12)	0.88(0.55,1.42)	1.11(0.81,1.52)	1.31(0.85,2.03)	0.88(0.54,1.45)	0.89(0.66,1.20)	0.78(0.52,1.18)	1.10(0.70,1.73)
2times	0.90(0.68,1.20)	0.83(0.56,1.24)	1.03(0.67,1.57)	1.07(0.80,1.43)	0.97(0.65,1.45)	1.20(0.75,1.91)	0.96(0.73,1.26)	1.06(0.72,1.55)	0.86(0.57,1.31)
0-1time	1.00	1.00	1.00	1.00	1.00	1.00	1.00	1.00	1.00
Patient behaviors									
**Patient trust**									
Very low	0.22(0.15,0.33)***	0.21(0.12,0.36)***	0.18(0.09,0.37)***	5.32(3.34,8.46)***	4.99(2.75,9.07)***	8.20(3.68,18.24)***	0.32(0.21,0.49)***	0.34(0.19,0.58)***	0.22(0.10,0.48)***
Lower	0.23(0.17,0.33)***	0.20(0.13,0.31)***	0.25(0.14,0.45)***	3.95(2.80,5.58)***	3.81(2.45,5.94)***	5.28(2.89,9.64)***	0.48(0.33,0.69)***	0.55(0.35,0.86)**	0.33(0.17,0.64)**
Average	0.37(0.27,0.51)***	0.31(0.21,0.46)***	0.39(0.22,0.67)***	1.89(1.39,2.57)***	1.93(1.30,2.85)***	2.36(1.36,4.09)**	0.70(0.50,0.99)***	0.72(0.47,1.11)	0.55(0.29,1.06)
Higher/Very high	1.00	1.00	1.00	1.00	1.00	1.00	1.00	1.00	1.00
**Unreasonable requests from patients**									
A lot	0.84(0.54,1.30)	0.88(0.48,1.60)	0.81(0.41,1.60)	3.37(1.70,6.69)***	3.31(1.40,7.82)**	2.78(0.86,8.97)	0.52(0.32,0.84)***	0.52(0.26,1.02)	0.44(0.21,0.92)*
More	0.70(0.55,0.90)***	0.71(0.51,0.99)*	0.62(0.41,0.92)*	1.53(1.18,1.98)***	2.04(1.44,2.91)***	1.14(0.75,1.72)	0.58(0.45,0.73)***	0.64(0.46,0.88)**	0.45(0.30,0.66)***
Average	0.82(0.66,1.02)	0.83(0.62,1.11)	0.79(0.55,1.14)	1.21(0.96,1.52)	1.21(0.91,1.63)	1.27(0.87,1.86)	0.73(0.58,0.91)***	0.89(0.66,1.19)	0.54(0.37,0.78)***
Less/Rarely	1.00	1.00	1.00	1.00	1.00	1.00	1.00	1.00	1.00

Note: All Models for total participants adjusted for socio-demographics (gender, age, marital status, education level, economic status and professional ranking), hospitals and department characteristics (hospital level, hospital type, academic status, physician specialty and the ratio of physicians to beds) and family support. The stratified analysis adjusted for all the covariates except for the stratified variable

*p<0.05

** *p*<0.01

*** *p*<0.001

In addition, significant gender differences were observed in the relationship between some organizational behaviors and physician well-being. Specifically, females who reported lower leadership attention to interests were more likely to have lower job satisfaction (OR _women_ = 0.20, 95% CI: 0.09–0.42) and higher career regret (OR _women_ = 4.73, 95% CI: 2.03–11.04). Male physicians who reported lower leadership attention to opinions were more likely to have higher career regret (OR _men_ = 2.29, 95% CI: 1.24–4.24). Having dinners with colleagues three times per month was associated with higher job satisfaction (OR _women_ = 2.28, 95% CI: 1.07–4.85) and higher level pf happiness (OR _women_ = 2.57, 95% CI: 1.10–6.02) of women physicians, while having dinners with colleagues ≥4 times was associated with lower career regret (OR _men_ = 0.49, 95% CI: 0.32–0.77) and higher level of happiness (OR _men_ = 2.07, 95% CI: 1.32–3.25) of men physicians.

## Discussion

To our knowledge, this study is the first study of physician well-being and its relevance to organizational and patient behaviors using a national representative sample in China. In general, more negative organizational behaviors and patient behaviors were positively associated with lower job satisfaction, lower level of happiness and higher career regret. Poor well-being and negative organizational and patient behaviors are common issues that confronts both male and female physicians in China, despite slight gender differences in various aspects.

These findings are relevant to current clinical practice and can apply to other countries, given the prevailing focus on physician well-being internationally. The current results confirm that the physician well-being should be approached from the perspective of internal and external environments. Whether organizational behaviors of the internal environment or patient behaviors of the external environment, healthcare providers (including hospital organizations and clinicians) can take action to change these behaviors, which can be modified to modify than structural factors of organization. The distribution of the exposure factors revealed that the proportion of positive responses was relatively low for both organizational and patient behaviors. Combined with the association of organizational and patient behaviors (reflecting the internal and external environment of hospital, respectively) with physician well-being, the current findings suggest that addressing these factors could substantially improve physician well-being.

Previous research [43, 44] indicates that low levels of organizational fairness can make employees feel excluded, potentially explaining the association of organizational fairness with physicians’ job satisfaction, level of happiness and career regret in this study. Specially, lower pay fairness was positively associated with higher career regret, and lower task fairness was positively associated with lower level of happiness. Prior work [45] indicates that, compared with distribution fairness [46] (reflected by pay fairness in this study), procedural fairness (reflected by task fairness in this study) may have a significant impact on people’s psychological health, attitudes and values, which is consistent with the results of this study.

In general, the mechanisms underlying the association between leadership attention and physician well-being by gender were less clear. Specially, lower leadership attention to interests was positively associated with lower job satisfaction and higher career regret of female physicians, and lower leadership attention to opinions was positively associated with higher career regret of male physicians. This gender difference may be related to traditional cultural difference between men and woman that men are concerned about their career while women are more concerned about their family responsivities. Additional studies will be required to explore this issue in more depth in future.

It may be unsurprising that more frequent clinical case discussions with colleagues per month was positively associated with higher job satisfaction which indicated, the important role of case discussions on professional development, which is not related to gender [47, 48]. However, having more dinners with colleagues per month were beneficial to both male and female physicians. Specially, having dinners with colleagues 3 times per month was associated with higher job satisfaction and higher level of happiness of women physicians, while having dinners with colleagues ≥4 times was associated with lower career regret and higher happiness of men physicians. This finding may have occurred because women tend to carry greater family responsibilities than men in China, having high-intensity social interaction may place a disproportionate burden on women [49, 50].

This study confirmed the important role of patient trust as there was a positive association between patient trust and physician well-being which was consistent with findings from previous studies [51–53].

Notably, China’s health care systems are based on public hospitals, and they follow the principles of cost control, efficiency improvement and patient-centered service, which are the same for countries with different health care systems around the world. Therefore, the lessons learnt from this study in relation to organizational behavior and patient behavior of public hospitals may be applicable to other countries.

As a strength of our study, the current analysis combined multiple organizational and patient behaviors, and the data was adjusted for a broad array of hospital organization and physicians’ demographic characteristics and family support factors known to influence physicians’ well-being. Importantly, our study involved several limitations. First, although we conducted a national survey, our study design did not enable us to identify differences between responding and non-responding physicians. Accordingly, the data was weighted by respondents’ age and gender to adjust for nonresponses. Second, data was self-reported by physicians. Thus, both underreporting and overreporting may have occurred, with the potential for variation in overreporting and underreporting regarding organizational and patient behaviors, as well as physician well-being, introducing uncertainty regarding the generalizability of our study findings. Third, given the busyness of participants’ job and the feasibility in a nationwide survey, the outcome variables in this study were measured using single-item indicators [5, 11, 32], and the measurement bias was possible. Fourth, the observational cross-sectional nature of the analyses precluded the ability to draw causal inferences. Nonetheless, the current study provided a large sample and multivariate evidence for in-depth investigation, which may enlighten future research.

## Conclusions

The national sample investigated physician well-being and its relevance to organizational behaviors and patient behaviors in China. In general, more negative organizational and patient behaviors (e.g., organizational fairness, leadership attention, team interaction and patient trust) were positively associated with lower physician well-being. The association between organizational behaviors and physician well-being exhibited some gender differences, while no clear gender difference was found for the relationship between patient behaviors and physician well-being. Given the prevailing international research interest in distress among physicians, interventions from the perspective of the internal and external hospital environment may provide a valuable approach for improving physician well-being.

## Supporting information

S1 File (DOCX)

## References

[1] Nojima Y, Kumakura S, Onoda K, Hamano T, Kimura K. Job and life satisfaction and preference of future practice locations of physicians on remote islands in Japan. Hum Resour Health. 2015;13:39. doi: 10.1186/s12960-015-0029-z . 26008624 PMC4446863

[2] Zhou H, Han X, Zhang J, Sun J, Hu L, Hu G, et al. Job satisfaction and associated factors among medical staff in tertiary public hospitals: results from a national cross-sectional survey in China. Int J Environ Res Public Health. 2018;15(7):1528. doi: 10.3390/ijerph15071528 . 30029506 PMC6068903

[3] Koch P, Zilezinski M, Schulte K, Strametz R, Nienhaus A, Raspe M. How perceived quality of care and job satisfaction are associated with intention to leave the profession in young nurses and physicians. Int J Environ Res Public Health. 2020;17(8):2714. doi: 10.3390/ijerph17082714 . 32326518 PMC7216191

[4] Goitein L. Physician well-being: addressing downstream effects, but looking upstream. JAMA Intern Med. 2014;174(4):533–534. doi: 10.1001/jamainternmed.2013.13253 . 24515255

[5] Verghese A. The importance of being. *Health Affairs*, 2016, 35(10):1924–1927. doi: 10.1377/hlthaff.2016.0837 27702966

[6] Friedberg MW, Chen PG, Van Busum KR, Aunon F, Pham C, Caloyeras J, et al. Factors affecting physician professional satisfaction and their implications for patient care, health systems, and health policy. Rand Health Q. 2014;3(4):1. . 28083306 10.7249/rhq-03-04-01 PMC5051918

[7] DeVoe J, Fryer GE Jr, Hargraves JL, Phillips RL, Green LA. Does career dissatisfaction affect the ability of family physicians to deliver high-quality patient care? J Fam Pract. 2002;51(3):223–228. 10.1016/S0095-4543(01)00004-5 . 11978232

[8] Shanafelt TD, Balch CM, Bechamps G, Russell T, Dyrbye L, Satele D, et al. Burnout and medical errors among American surgeons. Ann Surg. 2010;251(6):995–1000. doi: 10.1097/SLA.0b013e3181bfdab3 . 19934755

[9] Haas JS, Cook EF, Puopolo AL, Burstin HR, Cleary PD, Brennan TA. Is the professional satisfaction of general internists associated with patient satisfaction? J Gen Intern Med. 2000;15(2):122–128. doi: 10.1046/j.1525-1497.2000.02219.x . 10672116 PMC1495336

[10] Tak HJ, Curlin FA, Yoon JD. Association of intrinsic motivating factors and markers of physician well-being: a national physician survey. J Gen Intern Med. 2017;32(7):739–746. doi: 10.1007/s11606-017-3997-y . 28168540 PMC5481224

[11] Duffy RD, Manuel RS, Borges NJ, Bott EM. Calling, vocational development, and well being: A longitudinal study of medical students. J Vocat Behav. 2011,79(2):361–366. 10.1016/j.jvb.2011.03.023.

[12] Ryan RM, Deci EL. Intrinsic and extrinsic motivations: classic definitions and new directions. Contemp Educ Psychol. 2000;25(1):54–67. doi: 10.1006/ceps.1999.1020 . 10620381

[13] Scheurer D, McKean S, Miller J, Wetterneck T. U.S. physician satisfaction: a systematic review. J Hosp Med. 2009;4(9):560–568. doi: 10.1002/jhm.496 . 20013859

[14] VanLare JM, Blum JD, Conway PH. Linking performance with payment: implementing the physician value-based payment modifier. JAMA. 2012;308(20):2089–2090. doi: 10.1001/jama.2012.14834 . 23198298

[15] Kushnir T, Greenberg D, Madjar N, Hadari I, Yermiahu Y, Bachner YG. Is burnout associated with referral rates among primary care physicians in community clinics? Fam Pract. 2014;31(1):44–50. doi: 10.1093/fampra/cmt060 . 24148815 PMC5926437

[16] Landon BE, Reschovsky J, Blumenthal D. Changes in career satisfaction among primary care and specialist physicians, 1997–2001. JAMA. 2003;289(4):442–449. doi: 10.1001/jama.289.4.442 . 12533123

[17] Zumrah AR, Boyle S. The effects of perceived organizational support and job satisfaction on transfer of training. Pers Rev. 2015;44(2):236–254. 10.1108/PR-02-2013-0029.

[18] Kim A, Barak MM. The mediating roles of leader–member exchange and perceived organizational support in the role stress–turnover intention relationship among child welfare workers: a longitudinal analysis. Child Youth Serv Rev. 2015;52:135–143. 10.1016/j.childyouth.2014.11.009.

[19] Liu L, Wen F, Xu X, Wang L. Effective resources for improving mental health among Chinese underground coal miners: perceived organizational support and psychological capital. J Occup Health. 2015;57(1):58–68. doi: 10.1539/joh.14-0082-OA . 25410268

[20] Le Blanc PM, Hox JJ, Schaufeli WB, Taris TW, Peeters MC. Take care! The evaluation of a team-based burnout intervention program for oncology care providers. J Appl Psychol. 2007;92(1):213–227. doi: 10.1037/0021-9010.92.1.213 . 17227163

[21] Dunn PM, Arnetz BB, Christensen JF, Homer L. Meeting the imperative to improve physician well-being: assessment of an innovative program. J Gen Intern Med. 2007;22(11):1544–1552. doi: 10.1007/s11606-007-0363-5 . 17891503 PMC2219814

[22] Leitch KK, Walker PM. Surgeon compensation and motivation. Arch Surg. 2000;135(6):708–712. doi: 10.1001/archsurg.135.6.708 10843369

[23] Shanafelt T, Goh J, Sinsky C. The business case for investing in physician well-being. JAMA Intern Med. 2017;177(12):1826–1832. doi: 10.1001/jamainternmed.2017.4340 . 28973070

[24] Shanafelt TD, Gorringe G, Menaker R, Storz KA, Reeves D, Buskirk SJ, et al. Impact of organizational leadership on physician burnout and satisfaction. Mayo Clin Proc. 2015;90(4):432–440. doi: 10.1016/j.mayocp.2015.01.012 . 25796117

[25] Demmy TL, Kivlahan C, Stone TT, Teague L, Sapienza P. Physicians’ perceptions of institutional and leadership factors influencing their job satisfaction at one academic medical center. Acad Med. 2002;77(12 Pt 1):1235–1240. doi: 10.1097/00001888-200212000-00020 . 12480634

[26] Lin KY. Physicians’ perceptions of autonomy across practice types: Is autonomy in solo practice a myth? Soc Sci Med. 2014;100:21–29. doi: 10.1016/j.socscimed.2013.10.033 . 24444835 PMC3898537

[27] Montgomery AJ. The relationship between leadership and physician well-being: a scoping review. J Healthc Leadersh. 2016;8:71–80. doi: 10.2147/JHL.S93896 . 29355195 PMC5741010

[28] Kao AC, Jager AJ, Koenig BA, Moller AC, Tutty MA, Williams GC, et al; Physician perception of pay fairness and its association with work satisfaction, intent to leave practice, and personal health; *J Gen Intern Med*. 2018;33(6): 812–817. doi: 10.1007/s11606-017-4303-8 . 29380217 PMC5975140

[29] Zhang P, Wang F, Cheng Y, Zhang LY, Ye BZ, Jiang HW, et al. Impact of organizational and individual factors on patient-provider relationships: a national survey of doctors, nurses and patients in China. PLoS One. 2017;12(7):e0181396. doi: 10.1371/journal.pone.0181396 28753619 PMC5533441

[30] Díaz-Gracia L, Barbaranelli C, Moreno-Jiménez B. Spanish version of Colquitt’s Organizational Justice Scale. Psicothema. 2014;26(4):538–544. doi: 10.7334/psicothema2014.110 . 25340903

[31] Enoksen E. Examining the dimensionality of Colquitt’s Organizational Justice Scale in a public health sector context. Psychol Rep. 2015;116(3):723–737. doi: 10.2466/01.PR0.116k26w0 . 25871568

[32] West CP, Shanafelt TD, Kolars JC. Quality of life, burnout, educational debt, and medical knowledge among internal medicine residents. JAMA. 2011;306(9):952–960. doi: 10.1001/jama.2011.1247 . 21900135

[33] Dyrbye LN, Freischlag J, Kaups KL, Oreskovich MR, Satele DV, Hanks JB, et al. Work-home conflicts have a substantial impact on career decisions that affect the adequacy of the surgical workforce. Arch Surg. 2012;147(10):933–939. doi: 10.1001/archsurg.2012.835 . 23117833

[34] Rotenstein LS, Torre M, Ramos MA, Rosales RC, Guille C, Sen S, et al. Prevalence of burnout among physicians: a systematic review. JAMA. 2018;320(11):1131–1150. doi: 10.1001/jama.2018.12777 . 30326495 PMC6233645

[35] West CP, Dyrbye LN, Sloan JA, Shanafelt TD. Single item measures of emotional exhaustion and depersonalization are useful for assessing burnout in medical professionals. J Gen Intern Med. 2009;24(12):1318–1321. doi: 10.1007/s11606-009-1129-z 19802645 PMC2787943

[36] Shanafelt TD, Boone S, Tan L, Dyrbye LN, Sotile W, Satele D, et al. Burnout and satisfaction with work-life balance among US physicians relative to the general US population. Arch Intern Med. 2012;172(18):1377–1385. doi: 10.1001/archinternmed.2012.3199 . 22911330

[37] Lehmann-Willenbrock N, Meinecke AL, Rowold J, Kauffeld S. How transformational leadership works during team interactions: a behavioral process analysis. Leadership Quart. 2015;26:1017–1033. 10.1016/j.leaqua.2015.07.003.

[38] Laferton JA, Kube T, Salzmann S, Auer CJ, Shedden-Mora MC. Patients’ expectations regarding medical treatment: a critical review of concepts and their assessment. Front Psychol. 2017;8:233. doi: 10.3389/fpsyg.2017.00233 . 28270786 PMC5318458

[39] Swarup I, Henn CM, Gulotta LV, Henn RF. Patient expectations and satisfaction in orthopaedic surgery: a review of the literature. J Clin Orthop Trauma. 2019;10(4):755–760. doi: 10.1016/j.jcot.2018.08.008 . 31316250 PMC6611830

[40] Waljee J, McGlinn EP, Sears ED, Chung KC. Patient expectations and patient-reported outcomes in surgery: a systematic review. Surgery. 2014;155(5):799–808. doi: 10.1016/j.surg.2013.12.015 . 24787107 PMC4170731

[41] He G, An R, Zhang F. Cultural intelligence and work-family conflict: a moderated mediation model based on conservation of resources theory. Int J Environ Res Public Health. 2019;16(13):2406. doi: 10.3390/ijerph16132406 . 31284604 PMC6651476

[42] Guille C, Frank E, Zhao Z, Kalmbach DA, Nietert PJ, Mata DA, et al. Work-family conflict and the sex difference in depression among training physicians. JAMA Intern Med. 2017;177(12):1766–1772. doi: 10.1001/jamainternmed.2017.5138 . 29084311 PMC5820732

[43] Anderson N, Sinangil H, Viswesvaran C. Handbook of industrial, work and organizational psychology: some reflections on gorriti’s review. Int J Select Assess. 2004; 12(4):375–377. 10.1111/j.0965-075X.2004.00293.x.

[44] Ko J, Hur S. The impacts of employee benefits, procedural justice, and managerial trustworthiness on work attitudes: integrated understanding based on social exchange theory. Public Admin Rev. 2014;74(2):176–187. 10.1111/puar.12160.

[45] Tyler TR, De Cremer D. Process-based leadership: fair procedures and reactions to organizational change. Leadership Quart. 2005;16(4):529–545. 10.1016/j.leaqua.2005.06.001.

[46] Adams JS. Inequity in social exchange. Adv Exp Soc Psychol. 1965;2(4):267–299. 10.1016/S0065-2601(08)60108-2.

[47] Domagała A, Bała MM, Storman D, Peña-Sánchez JN, Świerz MJ, Kaczmarczyk M, et al; Factors associated with satisfaction of hospital physicians: a systematic review on European data; Int J Environ Res Public Health. 2018;15(11): 2546. https://www.ncbi.nlm.nih.gov/pmc/articles/PMC6266839/ doi: 10.3390/ijerph15112546 30428606 PMC6266839

[48] Mundt MP, Larissa I. Zakletskaia LI; professional communication networks and job satisfaction in primary care clinics; Ann Fam Med. 2019;17(5): 428–435. doi: 10.1370/afm.2442 . 31501206 PMC7032905

[49] Dyrbye LN, Freischlag J, Kaups KL, Oreskovich MR, Satele DV, Hanks JB, et al. Work-home conflicts have a substantial impact on career decisions that affect the adequacy of the surgical workforce. Arch Surg. 2012;147(10):933–939. doi: 10.1001/archsurg.2012.835 . 23117833

[50] Adám S, Gyorffy Z, László K. High prevalence of job dissatisfaction among female physicians: work-family conflict as a potential stressor. Orv Hetil. 2009;150(31):1451–1456. doi: 10.1556/OH.2009.28582 . 19617181

[51] Thom DH, Ribisl KM, Stewart AL, Luke DA. Further validation and reliability testing of the trust in physician scale. The stanford trust study physicians. Med Care. 1999;37(5):510–517. doi: 10.1097/00005650-199905000-00010 . 10335753

[52] Safran DG, Montgomery JE, Chang H, Murphy J, Rogers WH. Switching doctors: predictors of voluntary disenrollment from a primary physician’s practice. J Fam Pract. 2001;50(2):130–136. 10.1016/j.bjoms.2008.07.205 . 11219560

[53] Keating NL, Gandhi TK, Orav EJ, Bates DW, Ayanian JZ. Patient characteristics and experiences associated with trust in specialist physicians. Arch Intern Med. 2004;164(9):1015–1020. doi: 10.1001/archinte.164.9.1015 . 15136312

