# Supplementary material for: Association of organizational and patient behaviors with physician well-being: A national survey in China
Source: PLoS One. 2022 May 31;17(5):e0268274. doi: 10.1371/journal.pone.0268274 (PMC9154115; doi:10.1371/journal.pone.0268274)
Supplement: S1 File — (DOCX) [file pone.0268274.s001.docx]

**Supplementary Online Content**

**Association of Organizational and Patient Behaviors with Physician Well-being in China**

eFigure1. Flowchart for recruitment and response rates of the participants.

This supplementary material has been provided by the authors to give readers additional information about their work.

| **Sample of hospital** |  | **Hospitals selected** | | | | | | | | | |  |  |
| --- | --- | --- | --- | --- | --- | --- | --- | --- | --- | --- | --- | --- | --- |
|  |  | **Overall** | |  |  | **Hospital type** | |  |  | **Hospital level** | |  |  |
|  |  | 85 | |  |  | WM | TCM |  |  | TH | SH |  |  |
|  |  |  |  |  |  | 58 | 27 |  |  | 72 | 13 |  |  |
|  |  |  |  |  |  |  |  |  |  |  |  |  |  |
|  | Refused | Overall | =8(9.41%) |  |  | WM | =7(12.07%) |  |  | TH | =8(11.11%) |  |  |
|  |  |  |  |  |  | TCM | =1(3.70%) |  |  | SH | =0 |  |  |
|  |  | **Overall** | |  |  | **Hospital type** | |  |  | **Hospital level** | |  |  |
|  |  | 77  (90.59%) | |  |  | WM | TCM |  |  | TH | SH |  |  |
|  |  |  |  |  |  | 51  (87.93%) | 26  (96.30%) |  |  | 64  (88.89%) | 13  (100%) |  |  |
|  |  |  |  |  |  |  |  |  |  |  |  |  |  |
|  |  |  |  |  |  |  |  |  |  |  |  |  |  |
| **Sample of department** |  | **Departments selected** | | | | | | | | | |  |  |
|  |  | **Overall** | |  |  | **Hospital type** | |  |  | **Hospital level** | |  |  |
|  |  | 528 | |  |  | WM | TCM |  |  | TH | SH |  |  |
|  |  |  |  |  |  | 367 | 161 |  |  | 451 | 77 |  |  |
|  |  |  |  |  |  |  |  |  |  |  |  |  |  |
|  |  |  |  |  |  |  |  |  |  |  |  |  |  |
| **Sample of physicians** |  | **Physicians elegible** | | | | | | | | | |  |  |
|  |  | **Overall** | |  |  | **Hospital type** | |  |  | **Hospital level** | |  |  |
|  |  | 5754 | |  |  | WM | TCM |  |  | TH | SH |  |  |
|  |  |  |  |  |  | 4075 | 1679 |  |  | 4955 | 799 |  |  |
|  |  |  |  |  |  |  |  |  |  |  |  |  |  |
|  | Non-respondents | Overall | =1473(25.60%) | | | WM | =1064(26.11%) | | | TH | =1263(25.49%) | | |
|  |  |  |  | | | TCM | =409(24.36%) | | | SH | =210(26.28%) | | |
|  |  |  |  | | |  |  | | |  |  | | |
|  | Invalid questionnaires | Overall | =634(11.02%) | | | WM | =420(10.31%) | | | TH | =570(11.50%) | | |
|  |  |  |  | | | TCM | =214(12.75%) | | | SH | =64(8.01%) | | |
|  |  |  |  | | |  |  | | |  |  | | |
|  | Key research variables missing | Overall | =488(8.48%) | | | WM | =316 (7.75%) | | | TH | = 425(8.58%) | | |
|  |  |  |  | | | TCM | = 172(10.24%) | | | SH | = 63(7.88%) | | |
|  |  |  |  | | |  |  | | |  |  | | |
|  |  |  |  |  |  |  |  |  |  |  |  |  |  |
|  |  | **Overall** | |  |  | **Hospital type** | |  |  | **Hospital level** | |  |  |
|  |  | 3159  (54.90%) | |  |  | WM | TCM |  |  | TH | SH |  |  |
|  |  |  |  |  |  | 2275 | 884 |  |  | 2697 | 462 |  |  |
|  |  |  |  |  |  | (55.83%) | (52.65%) |  |  | (54.43%) | (57.82%) |  |  |

WM, Western Medicine. TCM, Traditional Chinese Medicine. TH, Tertiary Hospital. SH, Secondary Hospital.

eFigure1. Flowchart for recruitment and response rates of the participants.
