# Supplementary material for: Association of organizational and patient behaviors with physician well-being: A national survey in China
Source: PLoS One. 2022 May 31;17(5):e0268274. doi: 10.1371/journal.pone.0268274 (PMC9154115; doi:10.1371/journal.pone.0268274)
Supplement: Attachment — Submitted filename: PLOS ONE Review.docx [file pone.0268274.s002.docx]

My concern with this study is with the assessment tools that the authors used:

Overall, what do you think your happiness score is? – these are validated QoL measures that should have been used if the authors were interested in evaluating QoL

If you had an opportunity to choose your profession, would you become a physician again – this does not measure intention to leave/turnover but rather career regret

Leadership scores could have been assessed using validated tools

Team interaction being assessed by number of times that teams go out to dinner is highly subjective and likely not generalizable.

Unclear how patient behavior was assessed but these findings go against prior research (patient expectations and gender disparities in medicine).

Unclear how pay fairness in China is generalizable to other countries given the profoundly different pay structure
