# Supplementary material for: Association of organizational and patient behaviors with physician well-being: A national survey in China
Source: PLoS One. 2022 May 31;17(5):e0268274. doi: 10.1371/journal.pone.0268274 (PMC9154115; doi:10.1371/journal.pone.0268274)
Supplement: Attachment — Submitted filename: Response to Reviewers 2021-4-27.docx [file pone.0268274.s003.docx]

**[Response to the editor’s and reviewers’ comments](https://www.bmj.com/sites/default/files/attachments/bmj-article/pre-pub-history/Author%20response%2024.5.17.pdf)**

**Comments from the editor**

**Journal Requirements:**

When submitting your revision, we need you to address these additional requirements.

1. Please ensure that your manuscript meets PLOS ONE's style requirements, including those for file naming. The PLOS ONE style templates can be found at

https://journals.plos.org/plosone/s/file?id=wjVg/PLOSOne_formatting_sample_main_body.pdf and

[https://journals.plos.org/plosone/s/file?id=ba62/PLOSOne_formatting_sample_title_authors_affiliations.pdf](about:blank)

**Response:** We thank the editor for this comment and reminding; and we have done as suggested.

2. In the methods section please provide additional information regarding the following:
-    Please include additional information regarding the survey or questionnaire content validation.  
-    Please provide a justification for the sample size used in your study, including any relevant power calculations (if applicable
-    Details regarding the participant eligibility criteria
-    A description on how participants were recruited for the study
Furthermore, if the questionnaire is not under a copyright more restrictive than CC-BY, please include a copy, in both the original language and English, as Supporting Information.

**Response:** We thank the editor for this comment and reminding; and we have done as suggested.

**Reviewer #1:**

1. Overall, what do you think your happiness score is? – these are validated QoL measures that should have been used if the authors were interested in evaluating QoL.

**Response:** We greatly appreciated the above comments

At the beginning of the design, we prepared to adopt validated QoL measures, such as the Nottingham Health Profile, NHP; General Health Questionnaire, GHQ; however, the preliminary survey found that doctors in general hospitals are too busy and the feasibility for multi-items questionnaires is poor.

As a nationwide large sample survey, considering feasibility, we gave up using the validated QoL measures. On the basis of literature and expert consultation [32-36], especially with reference to related surveys (as the following) by American doctors, we adopted similar survey indicators and methods in the current study.

32.	West CP, Shanafelt TD, Kolars JC. Quality of life, burnout, educational debt, and medical knowledge among internal medicine residents. JAMA. 2011;306(9):952-960. <https://doi.org/10.1001/jama.2011.1247> PMID: 21900135

33.	Dyrbye LN, Freischlag J, Kaups KL, Oreskovich MR, Satele DV, Hanks JB, et al. Work-home conflicts have a substantial impact on career decisions that affect the adequacy of the surgical workforce. Arch Surg. 2012;147(10):933-939. <https://doi.org/10.1001/archsurg.2012.835> PMID: 23117833

34.	Rotenstein LS, Torre M, Ramos MA, Rosales RC, Guille C, Sen S, et al. Prevalence of burnout among physicians: a systematic review. JAMA. 2018;320(11):1131-1150. <https://doi.org/10.1001/jama.2018.12777> PMID: 30326495

35.	West CP, Dyrbye LN, Sloan JA, Shanafelt TD. Single item measures of emotional exhaustion and depersonalization are useful for assessing burnout in medical professionals. J Gen Intern Med. 2009;24(12):1318-1321. <https://doi.org/10.1007/s11606-009-1129-z> PMID: 19802645

36.	Shanafelt TD, Boone S, Tan L, Dyrbye LN, Sotile W, Satele D, et al. Burnout and satisfaction with work-life balance among US physicians relative to the general US population. Arch Intern Med. 2012;172(18):1377-1385. <https://doi.org/10.1001/archinternmed.2012.3199> PMID: 22911330

2. If you had an opportunity to choose your profession, would you become a physician again – this does not measure intention to leave/turnover but rather career regret.

**Response:** We thank the reviewer for this comment and have done as suggested (use “career regret” instead of “turnover intention”).

3. Leadership scores could have been assessed using validated tools.

**Response:** We greatly appreciated the above comments

In fact, the explanation and solution of the first question above also apply to this problem

4. Team interaction being assessed by number of times that teams go out to dinner is highly subjective and likely not generalizable.

**Response:** We greatly appreciated the above comments

Similarly, the explanation and solution of the first question above also apply to this problem

In fact, in existing related studies [37], the number of meetings is used as team interaction, similarly, in this study, number of dinners with colleagues per month (reflecting emotional interactions) and number of clinical case meetings per month (reflecting work interactions) were used as team interaction.

37.	Lehmann-Willenbrock N, Meinecke AL, Rowold J, Kauffeld S. How transformational leadership works during team interactions: a behavioral process analysis. Leadership Quart. 2015;26:1017–1033. <https://doi.org/10.1016/j.leaqua.2015.07.003>

5. Unclear how patient behavior was assessed but these findings go against prior research (patient expectations and gender disparities in medicine).

**Response:** We greatly appreciated the above comments

Different from some prior studies [38-40], patient behaviors in this study were measured from the physicians' perspective. Especially, the participants in this study were physicians, not patients, which may lead to these findings against some prior studies.

38.	Laferton JA, Kube T, Salzmann S, Auer CJ, Shedden-Mora MC. Patients' expectations regarding medical treatment: a critical review of concepts and their assessment. Front Psychol. 2017;8:233. <https://doi.org/10.3389/fpsyg.2017.00233> PMID: 28270786

39.	Swarup I, Henn CM, Gulotta LV, Henn RF. Patient expectations and satisfaction in orthopaedic surgery: a review of the literature. J Clin Orthop Trauma. 2019;10(4):755-760. <https://doi.org/10.1016/j.jcot.2018.08.008> PMID: 31316250

40.	Waljee J, McGlinn EP, Sears ED, Chung KC. Patient expectations and patient-reported outcomes in surgery: a systematic review. Surgery. 2014;155(5):799-808. <https://doi.org/10.1016/j.surg.2013.12.015> PMID: 24787107

6. Unclear how pay fairness in China is generalizable to other countries given the profoundly different pay structure.

**Response:** We greatly appreciated the above comments

The pay fairness mentioned in this study reflects distributive fairness, which refers to the distributive fairness of an organization, but does not involve the specific pay structure. Pay fairness would reflect the distributive fairness of an organization, which is of general concern to individuals, regardless of countries. Therefore, the result of this study would be of value to other countries.

**Reviewer #2:**

**[1] Major**

1. Pages 6-7. Statistical analysis and results

The authors conducted all analysis stratified by physician gender.

(1) Please clarify the logic underlying why the authors stratified the entire analyses by physician gender. For example, I was wondering if the authors assume physician gender is the most critical component that influence or differentiate physician well-being. Although some estimation results were different by physician gender, differences could be observed even when the authors stratified the analysis by other factors such as physician specialty, hospital level (secondary vs. tertiary), or hospital type (traditional Chinese medicine vs. western medicine).

**Response:** We greatly appreciated the above comments.

With the increase of double-income families, more and more women are entering the workforce, including in health care. Compared with men, women are a relatively disadvantaged group. A subset of studies investigating depression among training physicians have found higher rates among female physicians, and women are relatively more responsible at home, one factor that may affect female and male physicians differently is work-family conflict, where work obligations negatively impact family roles [41,42]. Therefore, from the perspective of gender stratification, this study analyzes the gender differences in the impact of organizational behavior and patient behavior on physician well-being.

41.	He G, An R, Zhang F. Cultural intelligence and work-family conflict: a moderated mediation model based on conservation of resources theory. Int J Environ Res Public Health. 2019;16(13):2406. <https://doi.org/10.3390/ijerph16132406> PMID: 31284604

42.	Guille C, Frank E, Zhao Z, Kalmbach DA, Nietert PJ, Mata DA, et al. Work-family conflict and the sex difference in depression among training physicians. JAMA Intern Med. 2017;177(12):1766-1772. <https://doi.org/10.1001/jamainternmed.2017.5138> PMID: 29084311

(2) If the table space allows, I recommend presenting estimation results among the total study population (in which physician gender and all explanatory variables are adjusted) and stratifying by physician gender.

**Response:** We greatly appreciated the above comments, and have presented estimation results among the total study population and stratifying by physician gender (the Tables 4 for details).

2. Page 7, fourth paragraph

I recommend deleting this paragraph and the associated results in the appendix table for two reasons:

(1) The authors have three co-primary outcomes (job satisfaction, turnover intention, life happiness). In their "sensitivity analysis," the same variable was used as an outcome variable in one estimation, but as an explanatory variable in another estimation (e.g., job satisfaction is an outcome variable, then becomes an explanatory variable for turnover intention). In statistics, this implies simultaneity between two outcomes, which would require a more advanced estimation model to generate unbiased estimates.

**Response:** We greatly appreciated the above comments, and have deleted in the sixth paragraph of the Results and the associated results in the appendix table.

(2) I am not sure whether “sensitivity analysis” usually refers to the adjustment of additional explanatory variable(s) while all other parts of the empirical design (e.g., applicable study population, categorization of outcome/independent variable) are the same.

**Response:** We greatly appreciated the above comments.

Sensitivity analysis is usually a way of testing the robustness of analysis results, including further adjusting for potential confounders, excluding participants, or adjust the type of outcome/independent variable(s).

Yu K, Qiu G, Chan KH, Lam KH, Kurmi OP, Bennett DA, et al. Association of solid fuel use with risk of cardiovascular and all-cause mortality in rural China. JAMA. 2018;319(13):1351-1361. <https://doi.org/10.1001/jama.2018.2151> PMID: 29614179

**[2] Minor**

1. P.6. Statistical analysis

The authors stated, “Data were weighted to adjust for non-responses so that physician responding to the initial questions were matched to the demographics of the total hospitalized population issued by the National General Hospital.” Please clarify whether this means that adjusting weight made the data nationally representative at the physician level.

**Response:** We greatly appreciated the above comments.

In fact, it is difficult to answer this question accurately. Low response rates are common in clinicians surveys all over the world. Doctors are busy and under big pressure, and their feasibility of taking part in the long survey is low, and correspondingly, the response rate is usually low. To address the question, we weighted the study sample according to the relevant statistical information of the doctor’s gender and age published in the 2015 China Health and Family Planning Statistical Yearbook.

2. P.7. Results section

(1) Please include the 95% confidence interval for each odds ratio (OR).

**Response:** We thank the reviewer for this comment and have included the 95% confidence interval for each odds ratio (OR) (the fourth and fifth paragraphs of the Results for details).

(2) Please specify whether the difference in association of organizational/patient characteristics with outcomes between men and women is a difference in magnitudes (i.e., OR) or statistical significance. Indeed, estimates show that some associations are statistically significant among men but not among women, and vice versa.

**Response:** We thank the reviewer for this comment. The association of the organizational/ patient characteristics with results between men and women is different in magnitudes (i.e., OR) or statistical significance. In fact, we have described it in the fifth paragraph of the Results. We have included the 95% confidence interval for each odds ratio (OR).

3. Please clarify some terminologies in the text. I have two examples:

(1) Page 7, second paragraph. The authors stated, “Table 4 …. after adjustment for potentially confounding factors.” I suggest replacing “potentially confounding factors” with “all other explanatory variables” because the term “confounding factors” usually means unobserved factors included in the error term, which are correlated with independent variables and cause bias in estimates.

**Response:** We thank the reviewer for this comment, and have revised “potentially confounding factors” into “all other explanatory variables”.

(2) Page 7, fourth paragraph. I am not sure whether “sensitivity analysis” is the correct terminology in this context.

**Response:** We thank the reviewer for this comment and have deleted the associated description and results of sensitivity analysis.
