# Supplementary material for: Association of organizational and patient behaviors with physician well-being: A national survey in China
Source: PLoS One. 2022 May 31;17(5):e0268274. doi: 10.1371/journal.pone.0268274 (PMC9154115; doi:10.1371/journal.pone.0268274)
Supplement: Attachment — Submitted filename: PONE-S-20-39067 Response to Reviewers 2022-2-26.docx [file pone.0268274.s005.docx]

**[Response to the editor’s and reviewers’ comments](https://www.bmj.com/sites/default/files/attachments/bmj-article/pre-pub-history/Author%20response%2024.5.17.pdf)**

**（February 26, 2020）**

PONE-D-20-31349R2

Association of Organizational and Patient Behaviors with Physician Well-being: A National Survey in China

**Journal Requirements:**

Please review your reference list to ensure that it is complete and correct. If you have cited papers that have been retracted, please include the rationale for doing so in the manuscript text, or remove these references and replace them with relevant current references. Any changes to the reference list should be mentioned in the rebuttal letter that accompanies your revised manuscript. If you need to cite a retracted article, indicate the article’s retracted status in the References list and also include a citation and full reference for the retraction notice.

**Response:** We thank the editor for this comment and reminding.

We did not cite papers that have been retracted in our manuscript.

Additional Editor Comments (if provided):

[1] Major comments. None

[2] Minor comments. Please make sure all terms and numbers are consistent throughout the abstract, text, and tables. For percentages, please write the first decimal point only (e.g., 11.02% - > 11.0%; both in text and tables).

**Response:** We thank the reviewers for this kind reminders, and we have done as suggested, and we have also further revised the English language.

**Reviewers' comments:**

**Comments to the Author**

1. If the authors have adequately addressed your comments raised in a previous round of review and you feel that this manuscript is now acceptable for publication, you may indicate that here to bypass the “Comments to the Author” section, enter your conflict of interest statement in the “Confidential to Editor” section, and submit your "Accept" recommendation.

Reviewer #3: All comments have been addressed

2. Is the manuscript technically sound, and do the data support the conclusions?

The manuscript must describe a technically sound piece of scientific research with data that supports the conclusions. Experiments must have been conducted rigorously, with appropriate controls, replication, and sample sizes. The conclusions must be drawn appropriately based on the data presented.

Reviewer #3: Yes

3. Has the statistical analysis been performed appropriately and rigorously?

Reviewer #3: Yes

4. Have the authors made all data underlying the findings in their manuscript fully available?

The [PLOS Data policy](http://www.plosone.org/static/policies.action#sharing) requires authors to make all data underlying the findings described in their manuscript fully available without restriction, with rare exception (please refer to the Data Availability Statement in the manuscript PDF file). The data should be provided as part of the manuscript or its supporting information, or deposited to a public repository. For example, in addition to summary statistics, the data points behind means, medians and variance measures should be available. If there are restrictions on publicly sharing data—e.g. participant privacy or use of data from a third party—those must be specified.

Reviewer #3: Yes

5. Is the manuscript presented in an intelligible fashion and written in standard English?

PLOS ONE does not copyedit accepted manuscripts, so the language in submitted articles must be clear, correct, and unambiguous. Any typographical or grammatical errors should be corrected at revision, so please note any specific errors here.

Reviewer #3: Yes

**Response:** We thank the reviewer for the positive comments.

**Reviewer #3:Comments to authors**

I appreciate the revisions from authors.

Minor

1. Table. Interaction

I would suggest modifying the wording in Table for readability. For example, Gender*Pay fairness -> Men*Pay fairness.

**Response:** We thank the reviewer for this kind reminders, and we have revised the wording in Table of the last response on November 1, 2021(at the end of this response).

2. The statement “Therefore, the study of organizational behavior and patient behavior of public hospitals in China may be universally applicable to other countries.”: This seems too generalizing the study findings. As the authors noted, the study effectively focuses on “organizational behavior” and “patient behavior” in public hospitals in one country. The findings could be relevant to some extent but should be interpreted with caution when it comes to different settings for example in other countries with healthcare systems that have a unique payment system, policy, regulation, culture,…...

**Response:** We thank the reviewer for this kind reminders, and we have revised as following:

The original

Therefore, the study of organizational behavior and patient behavior of public hospitals in China may be universally applicable to other countries.

The revised

Therefore, the lessons learnt from this study in relation to organizational behavior and patient behavior of public hospitals may be applicable to other countries.

3. 7. PLOS authors have the option to publish the peer review history of their article ([what does this mean?](https://journals.plos.org/plosone/s/editorial-and-peer-review-process#loc-peer-review-history)). If published, this will include your full peer review and any attached files.

If you choose “no”, your identity will remain anonymous but your review may still be made public.

**Do you want your identity to be public for this peer review?** For information about this choice, including consent withdrawal, please see our [Privacy Policy](https://www.plos.org/privacy-policy).

Reviewer #3: No

**Response:** We thank the reviewer for the constructive comments.

[**Response to the editor’s and reviewers’ comments**](https://www.bmj.com/sites/default/files/attachments/bmj-article/pre-pub-history/Author%20response%2024.5.17.pdf)

**（November 1, 2021: revised on February 26, 2020）**

**Comments from the editor**

PONE-D-20-31349R1

Association of Organizational and Patient Behaviors with Physician Well-being: A National Survey in China

PLOS ONE

Dear Dr. Liang,

Thank you for submitting your manuscript to PLOS ONE. After careful consideration, we feel that it has merit but does not fully meet PLOS ONE’s publication criteria as it currently stands. Therefore, we invite you to submit a revised version of the manuscript that addresses the points raised during the review process.

In addition to the reviewers’ comments, please note a few points:

1. The reviewers were concerned that some of the responses to the previous comments were not complete. Specifically, the discussion of the limitations in methods (e.g., validity of the variables) requires a clearer statement.

**Response:** We thank the editor for this comment and reminding; and we added the discussion of the limitations in methods as the following.

Third, given the busyness of participants’ job and the feasibility in a nationwide survey, outcome variables in this study were measured using single-item indicators[5,11,32], and the measurement bias is possible.

2. Please briefly discuss similarities and differences in (a) health care systems between China and other countries, and (b) study outcomes and policy implications from your study and relevant literature from other countries. This would be helpful to emphasize your contribution to the literature.

**Response:** We thank the editor very much for this very good revision comment; and we added the discussion of the limitations in methods as the following(This added content is placed before the strengths and limitations).

Notably, different from most other countries, China's health care systems are based on public hospitals, however, public hospitals in China also follow the principles of cost control, efficiency improvement and patient-centered service, which are the same for countries with different health care systems around the world. Therefore, the study of organizational behavior and patient behavior of public hospitals in China may be universally applicable to other countries.

3. The reviewer recommended including interaction terms between gender and other explanatory variables.

(a) I would recommend adding an interaction term between gender and one primary explanatory variable (e.g., hospital teaching status) per multivariable regression and checking whether the interaction term is statistically significant.

(b) Repeat (a) for a few explanatory variables that the authors believe are most important.

(c) If no interaction term is statistically significant, please summarize the results in a few sentences.

(d) If any interaction term is statistically significant, please

(d.i) derive adjusted predicted outcome, average marginal effect, and difference-in-differences given that interpretation of interaction term in a non-linear model is different from that in a linear model, and

(d.ii) report them in a few sentences (or the authors may want to write an appendix table).

**Response:** We thank the editor for the very good comments; and we have done as suggested.

Before gender stratification analysis, we did multivariable logistic regression for correlates of organizational and patient behaviors-related effects after increasing an interaction effect using gender with exposure factors, the results are as the following. The results showed that the combined effects of gender and exposure factors were in line with the multiplicative model, and there were negative (OR<1) OR positive (OR > 1) multiplicative interactions between gender and some exposure factors, which would indicate that physicians of different genders may have different influences on their well-being under different levels of exposure factors.

Table. Multivariable Logistic Regression Results for Correlates of Organizational and patient Behaviors-Related Effects after increasing an interaction effect using gender with exposure factors.

|  | **Job Satisfaction** |  | **Career Regret** |  | **Life Happiness** |
| --- | --- | --- | --- | --- | --- |
|  | **OR(95%CI)** |  | **OR(95%CI)** |  | **OR(95%CI)** |
| Gender*Pay fairness -> Men*Pay fairness | **0.74(0.58,0.95)**^*^ |  | 1.02(0.78,1.33) |  | 0.78(0.61,1.01) |
| Gender*Task fairness -> Men*Task fairness | 0.87(0.68,1.13) |  | 1.05(0.79,1.40) |  | 1.08(0.83,1.40) |
| Gender*Interests attention -> Men*Interests attention | 1.31(0.99,1.74) |  | **0.63(0.47,0.86)**^**^ |  | 1.04(0.78,1.38) |
| Gender*Opinions attention -> Men*Opinions attention | 0.89(0.67,1.18) |  | **1.47(1.09,1.99)**^*^ |  | 0.97(0.73,1.28) |
| Gender*The number of dinners with colleagues per month -> Men*The number of dinners with colleagues per month | 1.16(0.93,1.45) |  | 0.97(0.77,1.21) |  | **1.26(1.01,1.57)**^*^ |
| Gender*The number of clinical case discussions per month -> Men*The number of clinical case discussions per month | 0.99(0.85,1.16) |  | 1.15(0.98,1.36) |  | 0.88(0.75,1.02) |
| Gender*Patient trust -> Men*Patient trust | 1.04(0.82,1.32) |  | 0.86(0.66,1.12) |  | 1.17(0.92,1.50) |
| Gender*Unreasonable requests from patients -> Men*Unreasonable requests from patients | 0.95(0.76,1.17) |  | **1.36(1.07,1.73)**^*^ |  | 1.08(0.87,1.35) |

Note: All Models for total participants adjusted for socio-demographics (gender, age, marital status, education level, economic status and title), hospitals and department characteristics (hospital level, hospital type, academic status, physician specialty and the ratio of physicians to beds), family support and all exposure factors (pay fairness, task fairness, interests attention, opinions attention, the number of dinners with colleagues per month, the number of clinical case discussions per month, patient trust and unreasonable requests from patients).**p*<0.05, ** *p*<0.01, *** *p*<0.001

For example, the interaction item "Gender* interests attention" has an impact on the probability of physicians' High Career Regret, OR<1, The results showed that the influence of lower interests attention on the probability of High Career Regret among male doctors was weaker than those of female physicians. The interaction item "Gender* opinions attention" has an impact on the probability of physicians' High Career regrets, OR>1, which shows that the influence of lower opinions attention on the probability of High Career Regret among male physicians is stronger than those among female physicians. Therefore, in order to further analyze the specific gender differences in the impact of exposure factors on physicians’ well-being, we further adopted a gender-stratified analysis.

Additionally, as we have explained in the first revised response, with the increase of double-income families, more and more women are entering the workforce, including in health care. Compared with men, women are a relatively disadvantaged group. A subset of studies investigating depression among training physicians have found higher rates among female physicians. Therefore, from the perspective of gender stratification, this study analyzes the gender differences in the impact of organizational behavior and patient behavior on physician well-being.

[**Response to the editor’s and reviewers’ comments**](https://www.bmj.com/sites/default/files/attachments/bmj-article/pre-pub-history/Author%20response%2024.5.17.pdf)

**（November 1, 2021: revised on February 26, 2020）**

**Reviewer #3:**

Thank you for the opportunity to review your manuscript "Association of Organizational and Patient Behaviors with Physician Well-being: A National Survey in China". The physician's well-being is important for physicians themselves and the quality of care to patients. Please see my comments below. Thank you.

**Reviewer#1**

1. Comment 1 - This is a limitation of the study by not using validated measures of QoL.

**Response:** We thank the reviewer for this comment and reminding; and we added the discussion of the limitations in methods as the following.

Third, given the busyness of participants’ job and the feasibility in a nationwide survey, outcome variables in this study were measured using single-item indicators[5,11,32], and the measurement bias is possible.

2. Comment 4 has not been well addressed by the authors. The author-cited reference does not support the authors’ choice of the variable “number of times that teams go out to dinner”. The literature talks about various meetings in an organization, such as regular problem-solving team meetings, managerial meetings, or focus group meetings. These meetings are to facilitate interactions between team members and leadership in order to improve communication and coordination among members, and these activities are likely taking place in their regular work settings.

**Response:** We thank the reviewer for this comment and reminding and partially agree to the above recommendation.

As we have explained in the first revised response, in existing related studies[37], the number of meetings is used as team interaction, similarly, in this study, number of dinners with colleagues per month (reflecting emotional interactions) and number of clinical case meetings per month (reflecting work interactions) were used as team interaction.

Notably, the activities/meetings mentioned above (mainly reflecting work interactions) are likely taking place in their regular work settings, however, the activities/meetings of different team/group/organization is different, and individual participation in the team/group/organization is also different.

3. Comment 5 could be another limitation of the study as it did not capture patients’ perspectives directly from patients.

**Response:** We thank the reviewer for this comment and reminding and partially agree to the above recommendation.

Similar to the Hospital Consumer Assessment of Healthcare Providers and Systems (HCAHPS) survey, the survey of physicians’ and nurses’ behavior does not ask the doctor directly, but the patient.

During the hospital stay, how often did doctors listen carefully to you?

During the hospital stay, how often did nurses listen carefully to you?

4. Comment 6 – Given China-specific data and context, the findings are likely limited for other countries.

**Response:** We would like to thank the reviewer for the constructive comments; and we added the following in discussion.

Notably, different from most other countries, China's health care systems are based on public hospitals, however, public hospitals in China also follow the principles of cost control, efficiency improvement and patient-centered service, which are the same for countries with different health care systems around the world. Therefore, the study of organizational behavior and patient behavior of public hospitals in China may be universally applicable to other countries.

**Reviewer#2**

1. [2] Minor Comment 1 – The author’s response is still vague whether the estimation in the study is nationally representative by incorporating survey design features, such as stratification,…… Please clarify it.

**Response:** We would like to thank the reviewer for the constructive comments.

As we explained above, before gender stratification analysis, we did multivariable logistic regression for correlates of organizational and patient behaviors-related effects after increasing an interaction effect using gender with exposure factors, the results are as the following. The results showed that the combined effects of gender and exposure factors were in line with the multiplicative model, and there were negative (OR<1) OR positive (OR > 1) multiplicative interactions between gender and some exposure factors, which would indicate that physicians of different genders may have different influences on their well-being under different levels of exposure factors.

For example, the interaction item "Gender* interests attention" has an impact on the probability of physicians' High Career Regret, OR<1, The results showed that the influence of lower interests attention on the probability of High Career Regret among male doctors was weaker than those of female physicians. The interaction item "Gender* opinions attention" has an impact on the probability of physicians' High Career regrets, OR>1, which shows that the influence of lower opinions attention on the probability of High Career Regret among male physicians is stronger than those among female physicians. Therefore, in order to further analyze the specific gender differences in the impact of exposure factors on physicians’ well-being, we further adopted a gender-stratified analysis.

Table. Multivariable Logistic Regression Results for Correlates of Organizational and patient Behaviors-Related Effects after increasing an interaction effect using gender with exposure factors.

|  | **Job Satisfaction** |  | **Career Regret** |  | **Life Happiness** |
| --- | --- | --- | --- | --- | --- |
|  | **OR(95%CI)** |  | **OR(95%CI)** |  | **OR(95%CI)** |
| Gender*Pay fairness -> Men*Pay fairness | **0.74(0.58,0.95)**^*^ |  | 1.02(0.78,1.33) |  | 0.78(0.61,1.01) |
| Gender*Task fairness -> Men*Task fairness | 0.87(0.68,1.13) |  | 1.05(0.79,1.40) |  | 1.08(0.83,1.40) |
| Gender*Interests attention -> Men*Interests attention | 1.31(0.99,1.74) |  | **0.63(0.47,0.86)**^**^ |  | 1.04(0.78,1.38) |
| Gender*Opinions attention -> Men*Opinions attention | 0.89(0.67,1.18) |  | **1.47(1.09,1.99)**^*^ |  | 0.97(0.73,1.28) |
| Gender*The number of dinners with colleagues per month -> Men*The number of dinners with colleagues per month | 1.16(0.93,1.45) |  | 0.97(0.77,1.21) |  | **1.26(1.01,1.57)**^*^ |
| Gender*The number of clinical case discussions per month -> Men*The number of clinical case discussions per month | 0.99(0.85,1.16) |  | 1.15(0.98,1.36) |  | 0.88(0.75,1.02) |
| Gender*Patient trust -> Men*Patient trust | 1.04(0.82,1.32) |  | 0.86(0.66,1.12) |  | 1.17(0.92,1.50) |
| Gender*Unreasonable requests from patients -> Men*Unreasonable requests from patients | 0.95(0.76,1.17) |  | **1.36(1.07,1.73)**^*^ |  | 1.08(0.87,1.35) |

Note: All Models for total participants adjusted for socio-demographics (gender, age, marital status, education level, economic status and title), hospitals and department characteristics (hospital level, hospital type, academic status, physician specialty and the ratio of physicians to beds), family support and all exposure factors (pay fairness, task fairness, interests attention, opinions attention, the number of dinners with colleagues per month, the number of clinical case discussions per month, patient trust and unreasonable requests from patients).**p*<0.05, ** *p*<0.01, *** *p*<0.001

Additionally, as we have explained in the first revised response, with the increase of double-income families, more and more women are entering the workforce, including in health care. Compared with men, women are a relatively disadvantaged group. A subset of studies investigating depression among training physicians have found higher rates among female physicians. Therefore, from the perspective of gender stratification, this study analyzes the gender differences in the impact of organizational behavior and patient behavior on physician well-being.

**Additional comments**

**Abstract**

1. Given the study has been conducted in one country, the findings may not be generalizable to other countries, and so caution should be taken with stating policy implications.

**Response:** We would like to thank the reviewer for the constructive comments; and as we explained above, we added the following in discussion.

Notably, different from most other countries, China's health care systems are based on public hospitals, however, public hospitals in China also follow the principles of cost control, efficiency improvement and patient-centered service, which are the same for countries with different health care systems around the world. Therefore, the study of organizational behavior and patient behavior of public hospitals in China may be universally applicable to other countries.

**Introduction**

1. As the study is about physician well-being, not distress, the 1st sentence in the 1st paragraph could divert readers from the focus. It could be relocated elsewhere or removed.

**Response:** We would like to thank the reviewer for the constructive comments, and we have removed them as suggested.

2. In the 2nd paragraph, the authors limited the working environment factors to those (job autonomy, workload, scheduling…) and separated organizational behaviors (organizational fairness, team interaction, and so on). Whether they are job-specific factors or organizational behaviors, both constitute a working environment. I would suggest making sure consistency with the literature in using and defining these languages.

**Response:** We would like to thank the reviewer for this comments. This problem may be caused by our poor English ability, and we have barrier to accurately expressing our inner thoughts using non-native language.

In the 1st Paragraph, we have classified work environment factors as the internal and external hospital environments. Additionally, there are many environmental factors, as well as their classification, but these are not the focus of this study. In this study, we focused on a few modifiable internal environmental factors of hospital, such as organizational behavior.

3. In the same paragraph, a sentence starting with “Although previous studies have incorporated….”. Please cite a reference to support authors’ argument.

**Response:** We would like to thank the reviewer for the reminding, and we have done as suggested.

4. The next sentence starting with “compared with macro financial policy,..”. What is macro financial policy? And the whole sentence is not clear. Please rephrase it.

**Response:** We would like to thank the reviewer for the reminding, and we have done as the following:

Original

In addition, compared with macro financial policy, patients have the closest relationship to physicians, so modifying these factors may be easier for physicians.

Revised

In addition, compared with external financial policy, such as incentives or penalties from medical insurance agencies, patients have the closest relationship to physicians, so modifying these factors may be easier for physicians.

5. The sentence “types of organizational behaviors are relatively simple” needs supporting evidence.

**Response:** We would like to thank the reviewer for the reminding, and we have done as the following:

types of organizational behaviors are relatively simple, such as leadership and/or organization fairness [24,25,27,28],

6. Given this study is specific in China, more discussion of the background/context would be useful to help readers get better understanding. Also, I would suggest briefly discussing potential or expected contributions out of the study.

**Response:** We would like to thank the reviewer for the constructive comments; and as we explained above, we added the following in discussion.

Notably, different from most other countries, China's health care systems are based on public hospitals, however, public hospitals in China also follow the principles of cost control, efficiency improvement and patient-centered service, which are the same for countries with different health care systems around the world. Therefore, the study of organizational behavior and patient behavior of public hospitals in China may be universally applicable to other countries.

**Data collection**

1. In measuring team interaction, is there any justification/validation for including ‘number of dinners with colleagues per month’? This is quite subjective.

**Response:** We would like to thank the reviewer for the constructive comments; and as we explained above and the first response as the following.

In fact, in existing related studies [37], the number of meetings is used as team interaction, similarly, in this study, number of dinners with colleagues per month (reflecting emotional interactions) and number of clinical case meetings per month (reflecting work interactions) were used as team interaction.

**Statistical analysis**

1. Were survey design features accounted for in the analyses to make it nationally representative? If so, please state it.

**Response:** We would like to thank the reviewer for the constructive comments.

Just as we said in Study Design and Participants, “The details of this survey have been described in a previous report^[29]^”. Namely, the study was a stratified cluster sampling survey across the whole of mainland China. Provinces were selected from each geographical area, with two from each of East and West China (Shandong and Jiangsu, and Gansu and Yunnan), and one each from South, Central and North China (Guangdong, Hubei and Beijing metropolis), which have a population of 427.15 million, accounted for 31.88% of the total population of China. With reference to the 2013 and 2014 provincial GDP ranking, Guangdong and Beijing metropolis are representative of upper income areas; Gansu and Yunnan are representative of low income areas and the other three (Shangdong, Jiangsu and Hubei) are representative of middle income areas in China.

For the current study: A total of 5,754 physicians of 528 departments were asked to complete a survey. Participation was voluntary, and all data were confidential. 4281 (74.40% response rate) physicians responded to the survey and 634 (11.02%) invalid questionnaires were excluded. After excluding questionnaires missing key variables in this study, we selected a final study sample of 3159 physicians(54.90%) (See e Figure 1 in the Supplementary Appendix).

To adjust for nonresponses, the data was weighted by respondents’ age and gender, according to the hospitals’ demographic information issued by the National General Hospital in 2015.

2. When it comes to gender difference, what authors did seems to be a sub-group analysis within each gender. If authors intended to compare genders in the outcomes of interest as seen throughout the manuscript, I would recommend using an interaction effect using gender with other explanatory variables.

**Response:** We would like to thank the reviewer for the constructive comments.

As we explained above, before gender stratification analysis, we did multivariable logistic regression for correlates of organizational and patient behaviors-related effects after increasing an interaction effect using gender with exposure factors, the results are as the following. The results showed that the combined effects of gender and exposure factors were in line with the multiplicative model, and there were negative (OR<1) OR positive (OR > 1) multiplicative interactions between gender and some exposure factors, which would indicate that physicians of different genders may have different influences on their well-being under different levels of exposure factors.

For example, the interaction item "Gender* interests attention" has an impact on the probability of physicians' High Career Regret, OR<1, The results showed that the influence of lower interests attention on the probability of High Career Regret among male doctors was weaker than those of female physicians. The interaction item "Gender* opinions attention" has an impact on the probability of physicians' High Career regrets, OR>1, which shows that the influence of lower opinions attention on the probability of High Career Regret among male physicians is stronger than those among female physicians. Therefore, in order to further analyze the specific gender differences in the impact of exposure factors on physicians’ well-being, we further adopted a gender-stratified analysis.

Table. Multivariable Logistic Regression Results for Correlates of Organizational and patient Behaviors-Related Effects after increasing an interaction effect using gender with exposure factors.

|  | **Job Satisfaction** |  | **Career Regret** |  | **Life Happiness** |
| --- | --- | --- | --- | --- | --- |
|  | **OR(95%CI)** |  | **OR(95%CI)** |  | **OR(95%CI)** |
| Gender*Pay fairness -> Men*Pay fairness | **0.74(0.58,0.95)**^*^ |  | 1.02(0.78,1.33) |  | 0.78(0.61,1.01) |
| Gender*Task fairness -> Men*Task fairness | 0.87(0.68,1.13) |  | 1.05(0.79,1.40) |  | 1.08(0.83,1.40) |
| Gender*Interests attention -> Men*Interests attention | 1.31(0.99,1.74) |  | **0.63(0.47,0.86)**^**^ |  | 1.04(0.78,1.38) |
| Gender*Opinions attention -> Men*Opinions attention | 0.89(0.67,1.18) |  | **1.47(1.09,1.99)**^*^ |  | 0.97(0.73,1.28) |
| Gender*The number of dinners with colleagues per month -> Men*The number of dinners with colleagues per month | 1.16(0.93,1.45) |  | 0.97(0.77,1.21) |  | **1.26(1.01,1.57)**^*^ |
| Gender*The number of clinical case discussions per month -> Men*The number of clinical case discussions per month | 0.99(0.85,1.16) |  | 1.15(0.98,1.36) |  | 0.88(0.75,1.02) |
| Gender*Patient trust -> Men*Patient trust | 1.04(0.82,1.32) |  | 0.86(0.66,1.12) |  | 1.17(0.92,1.50) |
| Gender*Unreasonable requests from patients -> Men*Unreasonable requests from patients | 0.95(0.76,1.17) |  | **1.36(1.07,1.73)**^*^ |  | 1.08(0.87,1.35) |

Note: All Models for total participants adjusted for socio-demographics (gender, age, marital status, education level, economic status and title), hospitals and department characteristics (hospital level, hospital type, academic status, physician specialty and the ratio of physicians to beds), family support and all exposure factors (pay fairness, task fairness, interests attention, opinions attention, the number of dinners with colleagues per month, the number of clinical case discussions per month, patient trust and unreasonable requests from patients).**p*<0.05, ** *p*<0.01, *** *p*<0.001

Additionally, as we have explained in the first revised response, with the increase of double-income families, more and more women are entering the workforce, including in health care. Compared with men, women are a relatively disadvantaged group. A subset of studies investigating depression among training physicians have found higher rates among female physicians. Therefore, from the perspective of gender stratification, this study analyzes the gender differences in the impact of organizational behavior and patient behavior on physician well-being.

**Results**

1. In the 3rd paragraph, P-value in the text is different from the one in Table.

**Response:** We feel very guilty for our carelessness and we would like to thank the reviewer for the careful observation and kind comments, and we have done as suggested.

2. It would be more appropriate to use ‘association’ than ‘correlates’ for the title of Table 4.

**Response:** We would like to thank the reviewer for the careful observation and kind comments, and we have done as suggested.

3. In Table 4, please add statistical significance, such as using p-values. And, revise “for physicians” to “physician well-being”

**Response:** We would like to thank the reviewer for the careful observation and kind comments, and we have done as suggested.

4. Throughout the manuscript, be consistent by using “gender” to indicate men or women.

**Response:** We would like to thank the reviewer for the constructive comments., and we have done as suggested.

**Discussion**

1. In the 1st paragraph, the 2nd sentence can be separated, one for the overall association and the other one by gender.

**Response:** We would like to thank the reviewer for the constructive comments, and we have revised as the following.

**The original**

To our knowledge, the current study is the first national sample study of physician well-being and its relevance to organizational and patient behaviors in China. Despite gender differences in some aspects, poor well-being and negative organizational and patient behaviors are common issues that confronts physicians in China. In general, more negative organizational behaviors and patient behaviors were associated with lower job satisfaction and life happiness and higher career regret.

**The revised**

To our knowledge, this study would be the first national sample study of physician well-being and its relevance to organizational and patient behaviors in China. In general, more negative organizational behaviors and patient behaviors were associated with lower job satisfaction and life happiness and higher career regret. Despite gender differences in some aspects, poor well-being and negative organizational and patient behaviors are common issues that confronts physicians in China.

2. Delete current from “current findings”, and the phrase “which can be modified to some extent” is unclear.

**Response:** We would like to thank the reviewer for the constructive comments, and we deleted “current” from “current findings” and have revised “which can be modified to some extent” as “which may be more feasible to modify than structural factors of organization”.

3. In the 3rd paragraph, the 1st sentence would be part of study limitations “, and it is unclear about what the “psychological state” means and how the study findings are relevant to it.

**Response:** We would like to thank the reviewer for the constructive comments, and we deleted “the 1st sentence in the 3rd paragraph,” and have revised “psychological state” as “psychological health”.

4. In the 4th paragraph, the sentence starting with “It may be related….” seems to be not solely based on the study findings. For example, what authors say “women may be more concerned about their family” is not convincing.

**Response:** We would like to thank the reviewer for the constructive comments, and we have revised as the following.

**The original**

……, while women may be more concerned about their families, meaning that economic interests are relatively more important.

**The revised**

……, while women may be more concerned about their families, meaning that economic interests are relatively more important, especially for traditional Chinese women.

5. In the 5th paragraph, please cite relevant literature as necessary.

**Response:** We would like to thank the reviewer for the constructive comments., and we have done as suggested.

6. In the 6th paragraph, ‘association’ than ‘correlation’ would be a better term and be consistent across the manuscript.

**Response:** We would like to thank the reviewer for the constructive comments, and we have done as suggested.

7. In the 7th paragraph, “Compared” is inappropriate since it does not compare between the number of unreasonable requests from patients and patient trust. It could confuse readers. And, is there any justification for the interpretation in “A possible explanation…”? And the following sentence ‘patients’ distrust is not related to their medical knowledge” is unclear and unconvincing. Also, “excellent techniques” itself is confusing.

**Response:** We would like to thank the reviewer for this comments. The above issues may be caused by our poor English ability, as mentioned above, we have barrier to accurately expressing our inner thoughts using English language. We have revised the above issues as the following.

For “Compared” is inappropriate since it does not compare between the number of unreasonable requests from patients and patient trust. It could confuse readers.

**The original**

Compared with the number of unreasonable requests from patients, the current findings suggested that the correlation between patient trust and physician well-being was more important, given previous research about the important role of patient trust[49-51].

**The revised**

Compared with the association of unreasonable requests from patients for physician well-being, the current findings suggested that the association between patient trust and physician well-being was more important, given previous research about the important role of patient trust[51-53].

For And, is there any justification for the interpretation in “A possible explanation…”? And the following sentence ‘patients’ distrust is not related to their medical knowledge” is unclear and unconvincing. Also, “excellent techniques” itself is confusing.

**The original**

A possible explanation is that unreasonable requests from patients may be related to a lack of medical knowledge; patients may not intentionally make unreasonable requests, so the matter may be understandable and acceptable to physicians. However, patients’ distrust is not related to their medical knowledge, and may impact on physicians’ sense of dignity.

**The revised**

A possible explanation is that unreasonable requests from patients may be related to a lack of medical knowledge; patients may not intentionally make unreasonable requests, so the matter may be understandable and acceptable to physicians; however, patients’ distrust is not related to their medical knowledge, and may impact on physicians’ sense of dignity. **In other words, patients’ distrust may have more artificial or subjective hostility towards physicians than those of unreasonable requests.**

For Also, “excellent techniques” itself is confusing.

We have removed the sentence.
